# Prostate Cancer Detection with a Tactile Resonance Sensor—Measurement Considerations and Clinical Setup

**DOI:** 10.3390/s17112453

**Published:** 2017-10-26

**Authors:** Anders P. Åstrand, Britt M. Andersson, Ville Jalkanen, Börje Ljungberg, Anders Bergh, Olof A. Lindahl

**Affiliations:** 1 Department of Applied Physics and Electronics, Umeå University, SE-90187 Umeå, Sweden; britt.andersson@umu.se (B.M.A.); ville.jalkanen@umu.se (V.J.); 2 Centre for Biomedical Engineering and Physics, Umeå University, SE-90187 Umeå, Sweden; olof.lindahl@umu.se; 3 Department of Surgical and Perioperative Science, Urology and Andrology, Umeå University, SE-90187 Umeå, Sweden; borje.ljungberg@vll.se; 4 Department of Medical Biosciences, Pathology, Umeå University, SE-90187 Umeå, Sweden; anders.bergh@medbio.umu.se; 5 Department of Radiation Sciences, Biomedical Engineering, Umeå University, SE-90187 Umeå, Sweden

**Keywords:** tissue stiffness, resonance sensor, tactile sensor, prostate cancer, piezoelectric sensor

## Abstract

Tumors in the human prostate are usually stiffer compared to surrounding non-malignant glandular tissue, and tactile resonance sensors measuring stiffness can be used to detect prostate cancer. To explore this further, we used a tactile resonance sensor system combined with a rotatable sample holder where whole surgically removed prostates could be attached to detect tumors on, and beneath, the surface ex vivo. Model studies on tissue phantoms made of silicone and porcine tissue were performed. Finally, two resected human prostate glands were studied. Embedded stiff silicone inclusions placed 4 mm under the surface could be detected in both the silicone and biological tissue models, with a sensor indentation of 0.6 mm. Areas with different amounts of prostate cancer (PCa) could be distinguished from normal tissue (*p* < 0.05), when the tumor was located in the anterior part, whereas small tumors located in the dorsal aspect were undetected. The study indicates that PCa may be detected in a whole resected prostate with an uneven surface and through its capsule. This is promising for the development of a clinically useful instrument to detect prostate cancer during surgery.

## 1. Introduction

The use of a piezoelectric element as a resonance sensor for detecting tissue stiffness has been described already in the early 1990s [1]. Tactile resonance sensor systems based on the principle of an oscillating piezoelectric element, in contact with soft tissue, have been used to measure stiffness variations related to the heterogeneous prostate histology including malignant tissue [2,3]. In these studies, measurements were made on slices of a prostate gland. Tactile resonance sensors have also been used to measure differences in elasticity and stiffness to detect lesions and edema [4,5], liver fibrosis [6], and lymph node metastases [7].

New reliable and easy-to-use methods for early detection of clinically significant prostate cancer (PCa) are needed. PCa is the most common form of cancer among males in the Western world. The predicted number of deaths caused by PCa in 2016 was nearly 76,000 [8]. In Sweden, almost 11,000 new cases of PCa were diagnosed in 2014 and nearly 2400 men died, making Sweden a high-risk country for prostate cancer death [9]. The general trend since the late 1980s is an increase in PCa incidence, most likely due to an increased detection rate of latent disease using the blood test prostate specific antigen (PSA). In contrast to the incidence rise, the mortality rates have a decreasing trend in several countries, which may be due to an earlier detection of the disease [10].

The PSA test and digital rectal examination (DRE), when the physician palpates the prostate through the rectum, are the most common diagnostic methods used when PCa is suspected. The aim of the palpation is to detect stiff areas or nodules in the prostate, as it has been shown that tumors are usually stiffer, compared to healthy tissue [2,11,12]. In cases where the result of the DRE indicates PCa, microscopic evaluation of transrectal ultrasound (TRUS) guided needle-biopsies are used for diagnosis [13]. However, invasive biopsies also fail to detect 10–30% of PCa. Since the DRE are subjective and dependent on the physicians’ experience, an objective method and quantitative parameter related to the prostate tissue stiffness would be useful [14].

During minimally invasive surgery (MIS), assisted with robot technology or laparoscopy the surgeon can only feel the tissue through the instruments, which do not give much feedback regarding tissue composition [15]. There are recent studies on new techniques to improve the tactile feedback information from such instruments, also including the use of tactile sensors [15,16,17]. Tactile resonance sensors connected to the different surgical instruments might be a useful complement to assess tissue stiffness.

A tactile resonance sensor system (TRSS) used for the measurements in this study was presented earlier [18,19]. The measured parameters used for detecting differences in stiffness with the TRSS are the change in resonance frequency of the piezoelectric element, Δ*f*, and the applied force, *F*, during the indentation into the measured soft object. A stiffness parameter, |∂F/∂∆f| [20], could be obtained from the measured Δ*f* and *F* as functions of indentation depths, *I*. Through theoretical models, the stiffness parameter has been shown to relate to Young’s modulus, i.e., the elastic modulus of the measured object [20]. It has earlier been reported on the dependency of the parameters Δ*f*, *F*, and |∂F/∂∆f| on the contact angle, *α*, (i.e., deviation from perpendicular contact) indentation velocity, ν_i_ and *I* [18], as well as the depth sensitivity of |∂F/∂∆f| on flat tissue phantoms [19]. The results from these studies showed that a contact angle deviating ≤10° was acceptable for reliable measurements and that the detectable depth for the TRSS was 3.5 ± 0.5 mm. However, as a resected prostate gland has a spherical shape and is enclosed by a membrane, i.e., the capsule, new measurements on spherical objects are necessary before taking further steps towards a clinical application of the TRSS.

Previous studies have reported that prostate tumors often occur in the peripheral zone i.e., near the capsule [21,22]. When performing a radical prostatectomy, negative surgical margins is a prerequisite for optimal oncological results. Furthermore, the surgeon must avoid damaging of the neurovascular bundles to minimize the risk of future erectile problems for the patient. Therefore, it is important to investigate whether the tumor has penetrated the capsule and migrated into surrounding tissue, giving a positive surgical margin (PSM) [23,24,25]. Knowing the condition of the surgical margin gives the surgeon decision support as to whether or not to remove more tissue surrounding the prostate. One possible way to do that is to directly detect cancer on the surface of the prostate during surgery, as soon as the prostate is removed. This may be done by measuring the stiffness immediately on excised prostate with a tactile sensor and give decision support to the surgeon before closing up the surgery.

The aim of this study was to develop and evaluate a clinical TRSS setup enabling detection of cancer by measuring the stiffness on or close to the surface of surgically removed whole human prostate. Measurement considerations during application on prostate tissue and comparison with tissue phantoms as well as with golden standard histopathology were performed.

## 2. Materials and Methods

### 2.1. The Measurement System

**Figure 1:**
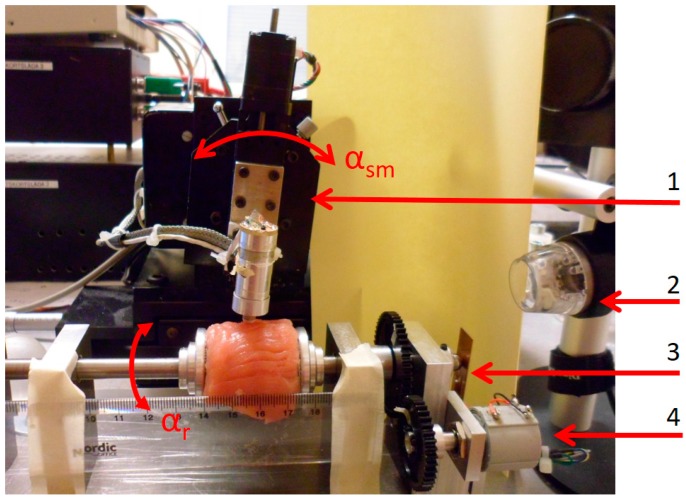
The sensor system set-up with a sample of porcine muscle mounted in the rotatable holder, ready for measurements. (1) The rotational stage to control the contact angle *α*. and the angle of the sensor movement, *α_sm_*; (2) USB-microscope; (3) The cantilever strain gauge; (4) A sensor for measuring the rotation angle *α_r_*.

The sensor probe used in this study has been described previously [18]. It consisted of a piezoelectric cylindrical element of lead zirconate titanate (PZT) (Morgan Electro Ceramics, Bedford, OH, USA). The cylinder was 15 mm long with the outer diameter of 5 mm and an inner diameter of 3 mm. The end of the piezoelectric element that contacts the measured object was made of polyether-ether-ketone (PEEK) in the shape of a hemisphere (a diameter of 5 mm). The resonance sensor and a preloaded force sensor (PS-05KC; Kyowa, Tokyo, Japan) were mounted inside an aluminium casing (Figure 1). The movement of the sensor was controlled and registered by an in-house developed application in LabView^®^ (National Instruments, Austin, TX, USA).

TRSS measurement of Δ*f* from the piezoelectric element and *F* from the force sensor were collected to a computer via a data acquisition card at a sampling rate of 1 kHz. The free (unloaded) resonance frequency was *f*_0_ = 113.8 kHz. The signal frequency was converted by a phase-locked-loop-circuit into a proportional DC-voltage prior to sampling with the data acquisition card. Thereafter, the frequency shift was calculated. From the indentation velocity the indentation depth, *I*, was calculated. In all measurements of this study, the total indentation depth *I_tot_* was 1.0 mm, and all data was analyzed at *I* = 0.6 mm. This depth was chosen as the stiffness parameter, |∂F/∂∆f| was calculated as the slope obtained from the change in Δ*f* and *F* during indentation. This was done through linear regression for an interval *I* = 0.6 ± 0.2 mm [19]. The indentation velocity was ν_i_ = 4 mm s^−1^.

To maintain a perpendicular contact angle *α*, between the moving direction of the sensor, and the tangent to the surface of the measured object, the sensor was rotated with an angle *α_sm_* (Figure 1). The TRSS had a rotatable holder with which a spherically shaped sample was rotated around its horizontal axis (Figure 1). An angle sensor measured the rotation angle, *α_r_*, relative to a reference point. The spherical sample was held in place by two spring loaded and adjustable concave aluminium discs. The clamping force, *F_C_*, by which the sample was held in place, was measured by a cantilever strain gauge. Both *α_r_* and *F_C_* were collected with the computer via the data acquisition card [19]. The measurement sequences were filmed or caught by snapshots through a USB microscope, (Dino-Lite AM4113TL, Dino-Lite Europe/IDCP B.V, Naarden, The Netherlands) as well as a digital camera (Samsung ST93, 16.1 megapixel, Elgiganten, Umeå, Sweden).

### 2.2. Measurements on Spherical Phantoms Made of Silicone

Silicones have been used as tissue phantoms for evaluating sensor techniques regarding human soft tissue characterization because of similar mechanical properties [3,26]. The silicone used in this study was of a two-component type (Wacker SilGel 612; Wacker-Chemie GmbH, Munich, Germany) [27], which has been used previously [2,26,28]. In this study, two different mixing ratios were used to obtain silicones with Shore hardness 33 and 88 (scale 000), according to ASTM D2240 [29]. The two mixtures were chosen to be in the same stiffness range as prostate tissue, both healthy and with tumors [3,30]. The relation between the mixing ratios, a stiffness value given by a standardized cone penetration values, and the stiffness parameter |∂F/∂∆f| can be found in [3]. Corresponding relations including the Shore hardness can be found in [30].

Three spherical silicone phantoms (Shore hardness 33) were cast using a mold with a diameter of 40 mm, as described in [30,31]. To simulate embedded stiffer nodules in soft tissue, inclusions of small spheres of silicone (Shore hardness 88) were made with diameters *D* = 2.5 mm, 4 mm, and 6 mm. One of the silicone spheres was homogeneous i.e., without inclusions and the other two contained three inclusions, placed along the circumference (great circle), at approximately 120° in between. The diameters of the four inclusions were *D* = 6 mm, and the others were *D* = 2.5 mm and *D* = 4 mm. The depths, *d*, (distance from the surface to the inclusion) were measured after all measurements were completed, by cutting the silicone spheres in halves. For the four inclusions with *D* = 6 mm, *d* were 0.2 mm, 0.5 mm, 3.7 mm, and 6.0 mm, respectively. For the other two, the inclusions with *D* = 4 mm and *D* = 2.5 mm, *d* were 2.2 mm and 1.6 mm. The procedure to position the stiffer nodules inside the mold has been described elsewhere [31]. The diameters of the nodules and depths were chosen to be close to the limit of detection for the 5 mm in diameter sensor tip used in this study [19].

**Figure 2:**
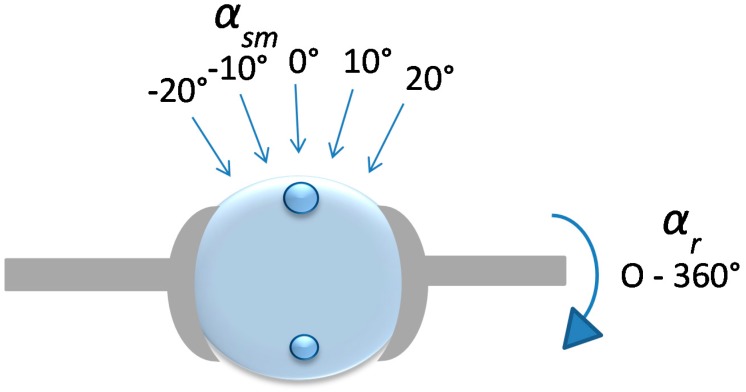
Illustration of a silicone sphere with embedded inclusions mounted in the rotatable sample holder. The movement of the sensor (not shown) for each MP was always kept perpendicular to the surface of the silicone. This was ensured by the rotation of the rotational stage by an angle *α_sm_*.

The silicone sphere without any inclusions was used to study the effect of the clamping force from the sample holder (see Section 2.4). For the silicon spheres with inclusions, the measurement positions (MPs) were chosen directly over the inclusions that corresponded to *α_sm_* = 0° and at positions at *α_sm_* = 10° and *α_sm_* = 20° axially, both to the left and right of the vertical (Figure 2). Measurements were also made in between the inclusions for reference. The measurements were repeated six times at each MP. For all measurements, the angle of the sensor was kept perpendicular to the surface at each MP using the rotational stage. The measurements were made at room temperature (21–23 °C), measured by a digital thermometer (Testo 623, Nordtec Instruments AB, Gothenburgh, Sweden).

### 2.3. Measurements on Phantoms Made of Porcine Tissue

Commercially available porcine muscle tissues from tenderloin were used as soft tissue phantoms to mimic human prostate tissue. Two samples were cut in sizes akin to that of the silicone spheres. The surfaces of the porcine muscles were cleaned and freed from visible membranes and tendons. One of the samples were used to study the clamping force (see Section 2.4). For the other sample, a small spherical silicone inclusion (Shore hardness 88) with a diameter of 6 mm was inserted, through an incision, in one of the tissue samples. The measurements were made following the circumference (the great circle) of the sample by rotating the tissue *α_r_* ± 35° from the position of the silicone inclusion in steps of Δ*α_r_* = 5°. The movement of the sensor was kept vertical for all measurements, i.e., *α* = *α_sm_* = 0°. One measurement was made at each MP. The measurements were made at room temperature (21–23 °C). The tissue was kept moist by spraying a saline solution on the surface with an atomizer before every measurement at approximately every fifth minute. After the measurements, the tissue was cut open and the distance, *d*, was measured.

### 2.4. Measurements of the Clamping Force

To examine how the clamping force from the sample holder, *F_C_*, affected the calculated parameter |∂F/∂∆f|, *F* and Δ*f* were measured as functions of *F_C_*, for both silicone and porcine tissue phantoms. An effect of the construction of the rotatable holder was that, as *F_C_* was increased, the center line of the measured sample was moved to the left towards the force sensor for *F_C_* (see Figure 1). For this reason, the position of the sensor had to be adjusted between each measurement to maintain its centered position. For the silicone, three repeated measurement series were performed on the homogeneous silicone sphere with *F_C_* ranging from 0 to approximately 2900 mN, with steps of 100 mN. The measurements were made at the same position with a minimum of 1 min in between. For porcine tissue, a minimum initial *F_C_* of 100 mN was necessary to keep the tissue sample from gliding since it was wet and slippery. To further prevent the tissue from slipping, small pieces of emery cloth was glued inside the concave discs. Four repeated measurement series were performed with *F_C_* up to approximately 2900 mN, with steps of 100 mN. In this case, the measurement positions were changed to avoid memory effects from a previous indentation. The time between measurements was at least 1 min.

### 2.5. Measurements on Human Prostate Glands Ex Vivo

**Figure 3:**
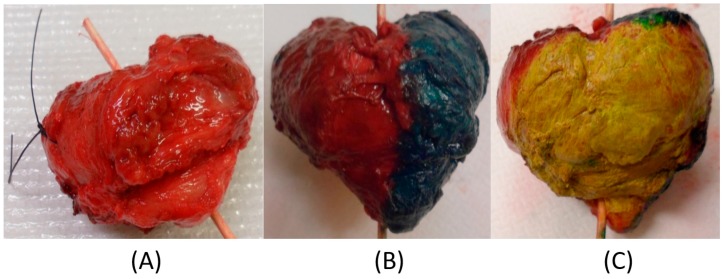
The resected radical prostatectomy specimen shortly after surgery from a 70-year-old man (Prostate 1 (P-1)). (**A**) Prostate gland without dye; (**B**) Dyed (green and red) on the (right and left) anterior side, apex is pointing down; (**C**) Dyed (yellow) on the dorsal side (the part facing the rectum).

Two prostate glands were obtained from patients, 70 and 72 years old, that were undergoing a radical prostatectomy at the University Hospital in Umeå. Both subjects gave their informed consent for inclusion before they participated in the study. The study was conducted in accordance with the Declaration of Helsinki, and the protocol was approved by the Ethics Committee at Umeå University (Dnr 03-423). The prostate glands of the younger man weighed 60.1 g and had the approximate diameter *D* = 50 mm (P-1). The other prostate gland weighed 40.6 g and had the approximate diameter *D* = 40 mm (P-2). After surgery, the prostate glands were stained according to the routines for histopathological procedures (yellow: dorsal aspect (backside); red: anterior (front) left side; green: anterior right side) before the measurements with the TRSS could commence (Figure 3).

The prostate glands were mounted with the urethra in the horizontal axial direction in the rotatable holder of the TRSS, approximately 40 min after removal from the patient due to logistic routines in the handling of the specimen. The pieces of emery cloth (see Section 2.4) were used inside the concave discs to ensure the glands were kept in position. Measurements were made at room temperature (21–23 °C). The prostate glands were rotated axially and measurements were made at every 10° (i.e., Δ*α_r_* = 10°) with the sensor in a vertical position, *α_sm_* = 0°. Close-up photographs of the sensor in contact with the prostate were taken from two angles at each measurement sequence. The measurements were made on all three regions that had been stained by the pathologist. The prostate glands were kept moist by spraying a physiological saline solution on the surface with an atomizer, approximately every fifth minute during the measurements. In a few cases, grooves on the surface of the prostate could hold excess saline solution, which then affected the measurement of *F*, but not Δ*f*, which caused |∂F/∂∆f| to approach zero and therefore be excluded. This phenomenon was also observed by Jalkanen et al. [32]. Such measurements were very few and for that reason omitted in this study.

A total of 36 and 41 measurements were made on Prostate 1 (P-1) and Prostate 2 (P-2), respectively, with 4.5 mm in between each MP. After the measurements, the line along the measurements was marked with spots of black marking dye. This was done to guide the pathologist when cutting the slices for histological analysis. These reference points were also an aid for locating the positions of the different MPs. For both prostates, a distinct point at the boundary of colors, and the corresponding *α_r_* were chosen as a starting point for the measurements. The maximum time the prostates were available for our measurements was 60 min. The measurements with the TRSS lasted 38 and 45 min, respectively, after which the prostate glands were returned to the pathologist for further processing and examination according to standard procedures.

The prostates were cut in transverse sections, approximately 0.5 cm thick, along the plane for the measurement points, dehydrated and embedded in paraffin. The sections were then cut in 5-μm-thick sections, stained with hematoxylin–eosin and examined with a light microscope according to routine histopathological procedures [33].

**Figure 4:**
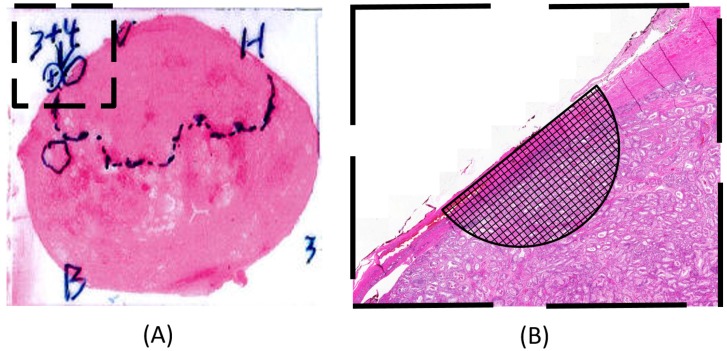
Illustration showing how the enlarged semi-circle together with the millimeter grid was used to estimate the proportion of tumor tissue within a given area near and below the surface for each measurement point. (**A**) A hematoxylin–eosin-stained tissue slice with the pathologists’ markings of areas with tumor tissue. The diameter of the prostate in the photomicrograph in (A) is approximately 40 mm; (**B**) A blow-up of the tissue area marked with a square in (A) for a certain measurement point in a high-resolution digital image.

The 5 μm sections were examined by a pathologist and photographed. The areas that showed tumor tissue were marked on the hematoxylin–eosin-stained photomicrograph (see Figure 4A). Each measurement point on the periphery of the prostate gland was identified on the photomicrograph. The composition of the tissue near and below each MP was investigated by determining the proportion of tumor tissue present, using a high-resolution digital image and a millimeter grid, shaped as a semi-circle, centered at each MP (see Figure 4B). The circle radius chosen corresponded to 3.5 mm on the prostate surface, and 1 mm in the grid corresponded to 0.15 mm. The dimension of the semi-circle was chosen as a result from a previous study [4] showing a depth sensitivity of 3.5 ± 0.5 mm for this TRSS set-up. The MPs were grouped according to the following occurrence of PCa: 0%; 1–30%; 31–60%; 61–100%.

### 2.6. Statistics

The measured values are presented as mean ± standard deviation (SD). Statistical testing for differences of the measurements on silicone were done with one-way ANOVA and a Tamhane’s post hoc multiple comparison tests. A Mann–Whitney–Wilcoxon test was used to test for differences between groups of the data from measurements on prostate tissue. Line fitting for the calculations of |∂F/∂∆f| from *F* and Δ*f* as well as evaluating the linear dependency between |∂F/∂∆f| and *F_C_* were done by linear regression. A Manova test was used for evaluating the occurrence of PCa at each MP. A *p*-value ≤ 0.05 was considered significant for all statistical tests used.

## 3. Results and Discussion

### 3.1. Spherical Phantoms Made of Silicone

**Figure 5:**
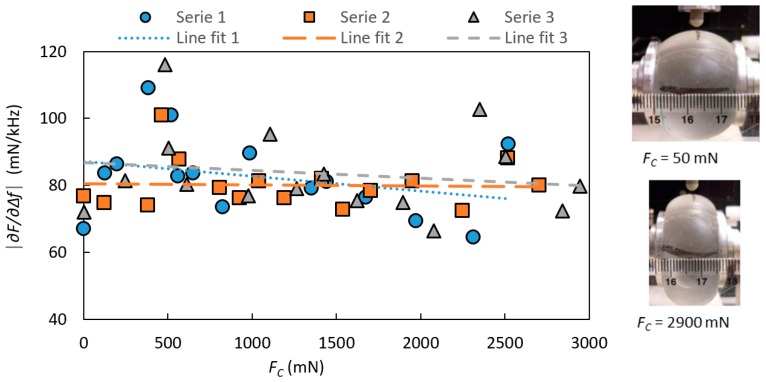
The stiffness parameter |∂F/∂∆f| with the indentation depth *I* = 0.6 mm, measured on a homogeneous silicone sphere as a function of the clamping force, *F_C_*. The measurements were made at an angle of sensor movement *α_sm_* = 0°. To the right, two pictures illustrate the visible effect of *F_C_* on the silicone sphere.

The effect of the clamping force *F_C_* on the stiffness parameter |∂F/∂∆f| is shown in Figure 5 for a silicone sphere for the three repeated measurement series. *F_C_* did not significantly change |∂F/∂∆f| as shown by linear regressions (R^2^ = 0.086, 0.002 and 0.030). For all further measurements on silicone spheres *F_C_* was kept at 500 ± 50 mN, which was sufficient to keep the silicone sphere securely in position.

Due to the construction of the rotatable holder, an increase in *F_C_* pushed the samples sideways (to the right, Figure 1, towards the force sensor for *F_C_* as explained in Section 2.4), which caused the center line of the sample to move as well. For this reason, the sensor was adjusted to maintain its centered position before each measurement. This could explain some of the variations in the measured |∂F/∂∆f| shown in Figure 5, as the silicone can show local stiffness variations [19].

**Figure 6:**
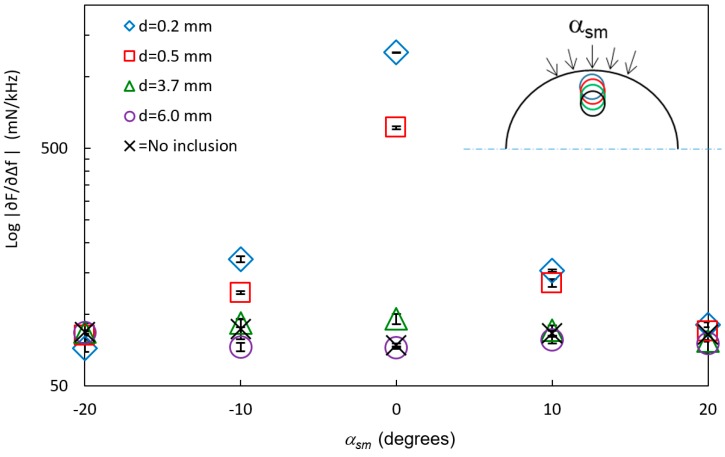
The logarithm of the stiffness parameter log|∂F/∂∆f| (mean ± SD, n = 6) at different angles of sensor movements *α_sm_* (see insert) on silicone spheres with inclusions with the diameter *D* = 6 mm but at different depths, *d*. Measurements made between the inclusions are marked “No inclusions”. The standard deviations (SD < 8.4 mN/kHz) are included in the graph.

In Figure 6, the stiffness parameter |∂F/∂∆f| is shown at different angles of sensor movements *α_sm_* for the four inclusions with a diameter *D* = 6 mm and at different depths from the surface, *d*. For comparison, reference measurements with no inclusions are also shown in the figure. The data show that the inclusions can be distinguished from the background when they are close to the surface. For *d* = 0.2 mm, the inclusion was discerned with a statistically significant (*p* < 0.05) difference from the soft silicone background for all angles. At *α_sm_* ± 20°, the inclusions at *d* = 0.5 mm, 3.7 mm, and 6.0 mm could not be detected from the soft silicone background (*p* > 0.05). The inclusions at *d* = 3.7 mm and 6.0 mm were detected at *α_sm_* = 0° and ±10° (*p* < 0.05).

At *α_sm_* = ±10°, the tip of the sensor, diameter 5 mm, still overlapped the inclusions as the distance between the MPs at *α_sm_* = 0° and *α_sm_* = ±10° on the surface of the mantle was 3.5 mm, and the sensor head was adjusted to keep a perpendicular indentation direction to the surface. The ability to detect the inclusions is a combination of both the amount of overlap between the sensor and the inclusion, in the direction of movement, and the increasing distance between the sensor and the inclusion, as the sensor is moved sideways. Earlier studies [19] pointed out a limitation regarding the detection of embedded silicone inclusions in flat surface silicone (silicones with the same shore hardness as in this study) to *d* = 3.5 ± 0.5 mm for the TRSS. The present study also showed a larger difference in |∂F/∂∆f| between silicone with and without stiffer inclusions. This can be explained by the fact that the inclusions in that study rested on the bottom of a Petri dish [19], whereas the inclusions in the present study did not have this solid support.

**Figure 7:**
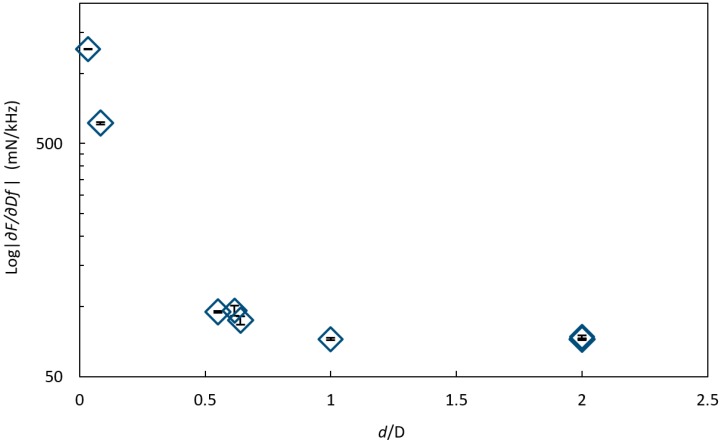
The logarithm of the stiffness parameter log|∂F/∂∆f| (mean ± SD, n = 6) against the ratio between the depths *d* of the inclusion and the diameter *D* of the inclusion, *d*/*D*, at *α_sm_* = 0° on inclusions of different sizes and at different depths in a silicon sphere showing that the inclusions could be detected up to about *d/D* = 0.6. The standard deviations are included in the graph.

The measured stiffness parameter |∂F/∂∆f| at *α_sm_* = 0° on all six inclusions of the two silicone spheres were compared by plotting log|∂F/∂∆f| against the ratio of *d*/*D* (Figure 7).

In this case, inclusions were detected up to about *d/D* = 0.6, meaning that large inclusions can be detected at a deeper depth *d* than the smaller inclusions. However, the signal from a small inclusion close to the surface cannot be distinguished from a larger inclusion further from the surface with the same ratio of *d/D*. This condition could be overcome in future applications by performing measurements in an array covering the area of interest and by combining the responses.

### 3.2. Phantoms Made of Porcine Tissue

**Figure 8:**
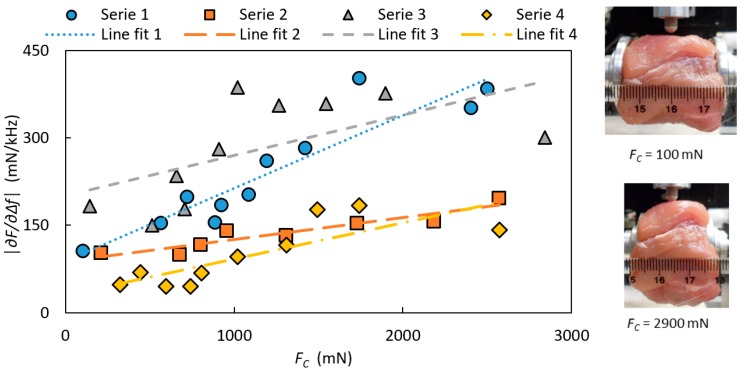
The stiffness parameter |∂F/∂∆f| for four separate measurement series as a function of the clamping force, *F_C_* measured on tissue from a porcine muscle with the indentation depth *I* = 0.6 mm and at the angle of sensor movement *α_sm_* = 0°. To the right, two pictures are illustrating the visible effect of *F_C_* on the tissue.

The effect on the stiffness parameter |∂F/∂∆f| due to the clamping force *F_C_* for porcine tissue for the four repeated measurement series are shown in Figure 8 with linear regressions.

For the measurements of the clamping force, the stiffness parameter |∂F/∂∆f| measured on porcine muscle increased when the clamping force *F_C_* was increased for all measurement series (Figure 7). A linear relation was fitted to the data, and all series showed a significant linear dependence (R^2^ = 0.8512, 0.8, 0.3673 and 0.6469) within the measured force range, but with different slopes. There was not a systematic change with time between the series, and as the surface was kept moist during the whole measuring sequence, this was expected. One explanation to the varying slopes could be that before each measurement series, the porcine muscle was put back in place in the holder, and this might have resulted in a small change in its position and thereby introduced a change of the internal pressure. This could also explain the different starting values for each series. One might guess that the relationship of |∂F/∂∆f| on *F_C_* is non-linear rather than linear over a wider force-range. However, holding a constant clamping force during measurements will ensure the ability to distinguish between tissues with different stiffness. The variation in |∂F/∂∆f| for the porcine tissue is higher than for the silicone sphere. The reason could be that for each measurement on the porcine tissue, the position of the MP was changed within an area of about 1 cm^2^, and the porcine muscle tissue could display variations in the mechanical properties. The surface was uneven compared to the silicone spheres, which might have resulted in an asymmetric contact surface, and therefore could affect the measured parameters. According to an earlier report [18], small variations in the contact angle (*α* < 10°) did not significantly affect the measured values of Δ*f* and *F* and thus the |∂F/∂∆f|. However, we can conclude that |∂F/∂∆f| was clearly affected (Figure 7) depending on how the porcine muscle sample was mounted and on the size of the clamping force. The purpose of the sensor is to differentiate between areas of different stiffness, and this will be done using a fixed clamping force.

**Figure 9:**
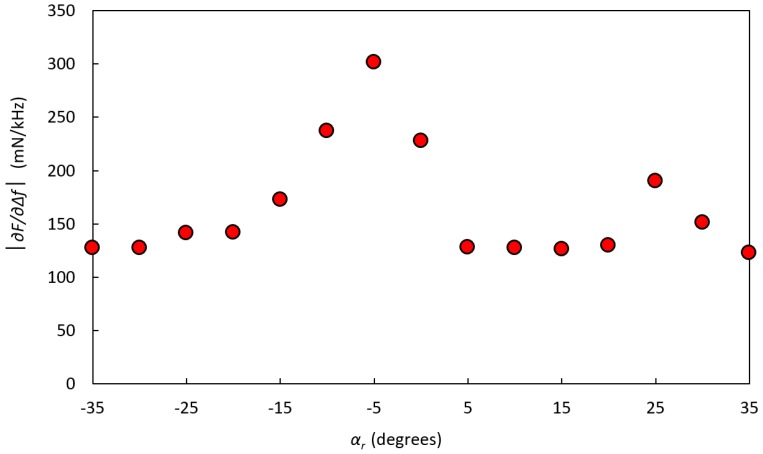
The stiffness parameter |∂F/∂∆f| on a sample of porcine muscle tissue with a hidden inclusion of silicone with Shore hardness 88, positioned at the rotation angle *α_r_* = −5° and located at a depth *d* = 3 ± 0.5 mm below the surface. The clamping force was *F_C_* = 425 ± 39 mN.

In Figure 9, the measured stiffness parameter |∂F/∂∆f| at indentation depth *I* = 0.6 mm for a porcine tissue sample with an embedded silicone inclusion at depth *d* = 3 ± 0.5 mm is shown. The inclusion could be detected at the rotation angle *α_r_* = −5°. At *α_r_* = +25°, another stiff area was observed, but this was shown to be a hidden tendon. A silicone inclusion embedded in the porcine muscle showed a larger contrast with the surrounding material compared to a similar inclusion in a silicone sphere. This larger contrast indicates that the system can more easily detect an inclusion in biological tissues than it can in silicone.

### 3.3. Measurements on Human Prostate Gland Ex Vivo

**Figure 10:**
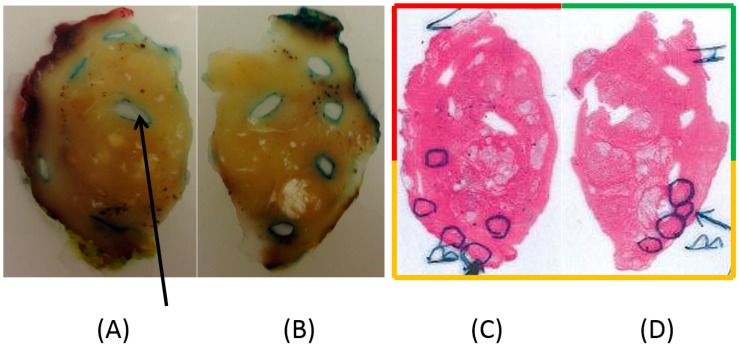
(**A**,**B**) Photographs of slices of the left and right sides of P-1, embedded in paraffin; (**C**,**D**) Scans of the corresponding photomicrographs. The original diameter of P-1 was 50 mm. Multiple areas of dorsal parts of the prostate identified as tumor tissue with a Gleason score of 6 (3 + 3) are marked with circles. The arrow in (**A**) shows one of the punch holes where tissue was removed after the stiffness analysis for further pathological assessments. In (**A**,**B**), the periphery of the sliced tissue shows traces of the red, green, and yellow staining of the surface made by the pathologist. The frame surrounding (**C**,**D**) is colored red, green, and yellow correspondingly.

For both prostates, the clamping force FC was chosen as low as possible to keep the prostates steady in place, which was less than, but close to, about 800 mN. P-1 is shown in Figure 10. Due to the size of P-1, a diameter of 50 mm, it was cut in two halves by the pathologist. The photomicrographs in Figure 10C,D show the tissue sections after treatment with hematoxylin–eosin, where areas with the presence of PCa were identified and marked by the pathologist.

**Figure 11:**
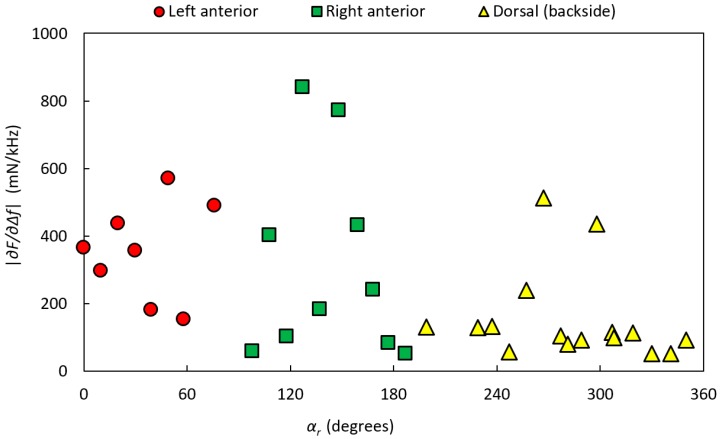
The stiffness parameter |∂F/∂∆f| of P-1 as a function of the rotational angle, *α_r_*, along the circumference of the gland. The prostate was clamped in the rotatable holder with the clamping force *F_C_* = 760 ± 99 mN. The data are divided into dorsal, left anterior, and right anterior according to the dyed areas made by the pathologist.

Figure 11 shows the stiffness parameter |∂F/∂∆f| values for P-1, divided into dorsal, left anterior, and right anterior according to the dyed areas made by the pathologist. Estimations of the amount of PCa from the high-resolution images for P-1 resulted in 24 MPs with 0% PCA, 9 MPs with 1–30% PCa, and 1 MP with 31–60% PCa. No MP had PCa >60%. All MPs with PCa were located in the dorsal aspect. MPs with PCa were compared with MPs from both the anterior and dorsal aspects with normal tissue, as well as from the dorsal aspect separately, for the three measured parameters, |∂F/∂∆f|, frequency shift Δ*f*, and contact force *F*. However, the analysis could not significantly differentiate between the groups for P-1.

Since the ability to identify PCa depends on the elastic contrast of the tumor compared with the surrounding tissue, false positives or false negatives can occur. The statistical analysis of the measurements on P-1 did not significantly differentiate between PCa and normal tissue. The PCa tissue was exclusively situated in the dorsal aspect of P-1 (see Figure 10C,D). The MPs only represented the 0% PCa group and the 1–30% PCa group, and one MP represented the 31–60% PCa group. A closer analysis of the high-resolution images showed that the PCa tissue appeared as small areas, 0.2–4 mm in diameter. A relationship between the Gleason score and the ability to detect tumors using real-time elastography has been reported, where a lower Gleason score resulted in a lower detection rate [34,35,36]. Langer et al. [37] pointed out that PCa can be of either dense or sparse histological architecture and showed that PCa with a Gleason score of 6 (3 + 3) are sparse with a mixture of normal and cancerous tissue, or includes glands with dilated lumina and are therefore soft. Some studies also indicates that elastography is more effective for the detection of tumors in the anterior region compared to the peripheral region [34,36].

There was no PCa in the anterior region of P-1, but some of the MPs showed elevated values of the stiffness parameter |∂F/∂∆f| indicating a stiffer tissue. The anterior region consists of more muscular tissue than other parts of the prostate, normally making this region stiffer. False positives could arise from the difference in stiffness between smooth muscle, connective tissue, glandular tissue [33], benign prostatic hypotrophy (BPH) [36,38], and calcifications. The MPs with the highest stiffness values in the dorsal region of P-1 were confirmed to contain calcifications.

**Figure 12:**
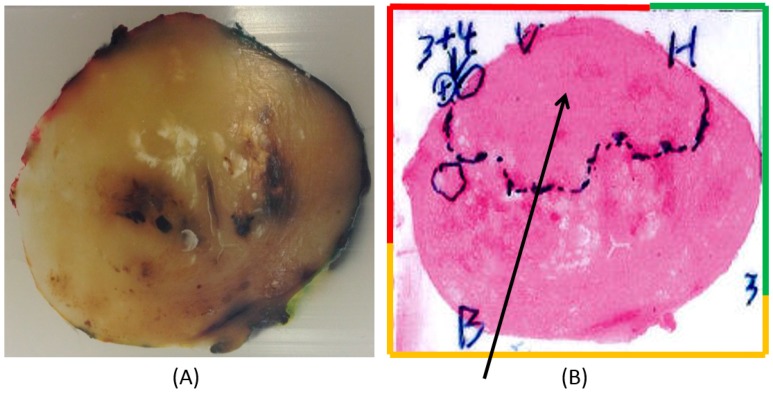
A slice of Prostate 2 (P-2). The diameter of P-2 was 40 mm. (**A**) A slice embedded in paraffin and stained at the periphery in order to locate the left anterior (red), right anterior (green), and dorsal (yellow) parts of the prostate. (**B**) Scanned photomicrograph of a section of the corresponding prostate slice. The arrow points out a large area marked by the pathologist in the anterior aspect containing cancer. In the left anterior, a tumor with a Gleason score of 7 (3 + 4) is also pointed out.

Prostate 2 (P-2) is shown in Figure 12A,B. The pathologist has marked a large area in the anterior region with a presence of PCa (Figure 12B).

**Figure 13:**
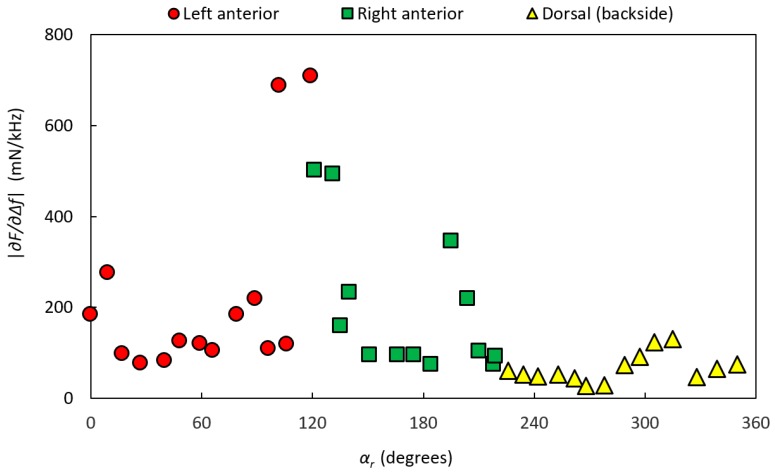
The stiffness parameter |∂F/∂∆f| of P-2 as a function of the rotational angle, *α_r_*, along the circumference of the gland. The prostate gland was clamped with the clamping force *F_C_* = 460 ± 85 mN in the rotatable holder. The data are divided into dorsal, left anterior, and right anterior according to the dyed areas made by the pathologist.

Figure 13 shows the stiffness parameter |∂F/∂∆f| values for P-2, divided into dorsal, left anterior, and right anterior according to the dyed areas made by the pathologist. For P-2, the analysis of high-resolution images resulted in 24 MPs with 0% PCA, 5 MPs with 1–30% PCa, and 12 MP with 61–100% PCa. No MPs were found in the interval 31–60% PCa. MPs with PCa were located in the anterior aspect.

**Figure 14:**
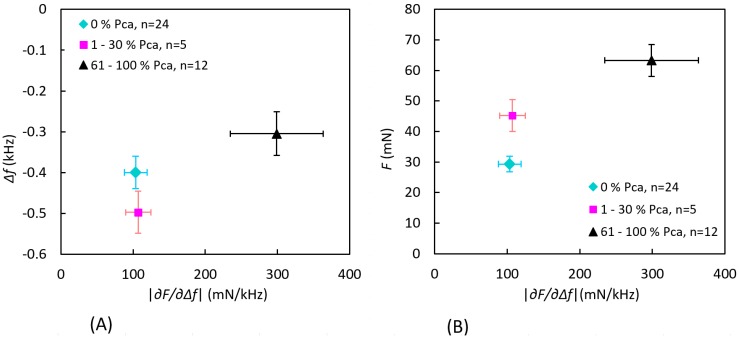
Measured values grouped by the amount of PCa occurrence within a semicircle with a radius of 3.5 mm at each measurement point for P-2. The data show the mean value of the stiffness parameter |∂F/∂∆f| ± standard error of the mean as a function of (**A**) the frequency shift Δ*f* and (**B**) the contact force *F*. There were no findings in the interval 31–60% PCa.

The MPs were grouped according to the amount of PCa as described earlier. To differentiate the groups, |∂F/∂∆f| was plotted as functions of Δ*f* and *F* as shown in Figure 14. Separations between the three groups were tested for significance using Manova, using all three parameters (Δ*f*, *F*, and |∂F/∂∆f|) as well as two parameters (Δ*f* and |∂F/∂∆f| or *F* and |∂F/∂∆f|). The tests gave the result *p* < 0.05, except for the separation of |∂F/∂∆f| for the 0% PCa and 1–30% PCa groups.

In P-2, the pathologist initially marked a large area containing PCa, concentrated to the anterior aspect (Figure 12B). A closer analysis of the high-resolution images confirmed that the PCa tissue was exclusively situated in this area. The three categorized groups of MPs could be significantly differenciated using two or all three parameters, but |∂F/∂∆f| alone could not differentiate between the 0% PCa group and the 1–30% PCa group. All MPs, i.e., data from both the anterior and dorsal aspects, were included in the comparison. Low stiffness values of MPs in the 1–30% PCa group can be explained by either very low PCa content or by the fact that the cancerous tissue was located well below the surface (still within the 3.5 mm radius). In addition, there were only five MPs in this group.

### 3.4. Uncertainty Analysis

Although pen markings were made and photos were taken at each measurement point, it was not trivial to subsequently identify the measurement points exactly in the high-resolution images of analyzing tissue content. The shape and proportions of the tissue sample may have changed due to the cutting and dehydration prior to the pathology analysis. Furthermore, P-1 was divided into two parts due to the size, and thus the shape was changed significantly near the cut. This may have caused some discrepancy between actual and estimated MPs. Moreover, for P-1, one out of the four spots of black dye (see Section 2.5) marking the line of measurements could not be found on the cut slice shown in Figure 10. This may indicate that the slice was cut with a possible deviation of a few millimeters on part of the left side of the prostate. However, through a number of fixed points and the fact that the precise Δ*α_r_* between each MPs were logged, the majority of the measurement points could be located with high precision on the high-resolution images. A more significant source of error could be that the pathological analysis was made on one cut transverse section, meaning that one two-dimensional surface would represent the three-dimensional volume under each MP.

Another source of error is the fact that the amount of PCa was only calculated within the gridded semi-circular areas, but there were no considerations about the actual locations of the PCa tissue within these areas. In addition, it is possible that PCa could have been located outside the gridded area and thus affected the stiffness value.

We would also like to point out that, for future clinical applications, it is the deviation in the parameters |∂F/∂∆f|, Δ*f*, and *F*, because of the PCa or the surrounding normal tissue within each individual prostate, that has to be considered.

The error estimates for the TRSS have been reported in previous studies [18,19]. The relative error, *ε*, for the indentation depth *I* at the indentation velocity ν_i_ = 4 mm s^−1^ was 3%. For the measured parameters Δ*f* and *F*, the relative *ε* was estimated to be <1.5% [36], as well as for the |∂F/∂∆f|. The relative *ε* for the strain gauge for measuring the clamping force *F_C_* was <0.2%, but due to friction in the mechanical construction the estimated *ε* for lower *F_C_* was about 10%. The *ε* for the angle sensor, *α_r_*, and the rotational stage, *α_sm_*, were ±0.5°. It has also been shown [18] that the angle of contact, *α*, is a source of error in the measurements of Δ*f* and *F* when the deviation from the perpendicular exceeds *α* > ±10°. For measurements made on biological tissue, which often have uneven surfaces, this has to be considered. In this study, photographs were taken just as the sensor made contact with the surface. The photographs were later studied, and *α* values were estimated to be less than 10° for all MPs. Therefore, in this study, no measurements had to be discarded due to a large *α*.

### 3.5. General Discussion and Conclusions

Novel results on TRSS measurements on whole human prostate glands are presented in the present study. The TRSS was used as an instrument aimed for detection of prostate cancer in surgically removed prostates. The results show that tumors located on or near the surface of a prostate ex vivo can be detected by the stiffness parameter |∂F/∂∆f| derived from the TRSS. Tactile stiffness measurements on whole human prostate glands have not been reported earlier. As the resected prostate tissue samples were subjects for pathological diagnostic analysis, the available time for our measurements in this study was limited to less than one hour. In order to make time consuming testing of the instrument in order to set optimal measurement parameters, the phantom materials were used. Tissue phantoms made as spheres of soft silicone as well as porcine muscle tissue were used for evaluation of the TRSS. The ability to detect inclusions made of silicone at different depths from the surface in both the silicone and porcine tissue phantoms was evaluated.

The present study was limited to ex vivo measurements on prostate but could be generalized also for in vivo measurements in e.g., robot-assisted surgery, giving haptic feedback to the surgeon. The PZT sensor used in the study could easily be miniaturized and introduced in a laparoscopic device. For example, a catheter-type resonance sensor with a diameter as small as 1.2 mm has been produced [26]. However, already in the present setting, the TRSS may be used to quickly determine the surgical margin during surgery, giving the surgeon a decision support whether or not to remove more surrounding tissue due to a positive surgical margin. This kind of decision support had not been obtainable without having a pathologist and a pathology lab standing by for histopathology.

In summary, in the prostate with more developed PCa, exclusively in the anterior, we could separate normal tissue from tissue containing different amounts of PCa. A rotatable holder worked effectively to scan different sides of the prostate. It was concluded that a varying clamping force of the rotatable holder could affect the stiffness measurements of biological tissue.

In conclusion, our novel study shows that PCa can be detected in a single whole resected prostate with an uneven surface and through its capsule, using TRSS. Future studies on whole prostates are wanted. A denser array of MPs, different sensor tip sizes, and larger indentation depths might contribute to the development of a method towards a clinically useful instrument to detect superficial prostate cancer.

## Abbreviations

CT	computed tomography
*D*	diameter
DRE	digital rectal examination
PCa	prostate cancer
MIS	minimally invasive surgery
MP	measurement position
PLL	phase-locked loop
PSA	prostate specific antigen
PSM	positive surgical margin
PZT	lead zirconate titanate, referring to the piezoelectric element
SD	standard deviation
TRSS	tactile resonance sensor system
TRUS	transrectal ultrasound
*α*	contact angle between sensor and object
*α_r_*	rotation angle
*α_sm_*	angle for sensor movement
Δ*α_r_*	step size between rotation angles
Δ*α_sm_*	step size between angles of sensor movement
*d*	distance to the surface above center of the stiff inclusion
ε	error
Δ*f*	frequency shift
*f*	loaded resonance frequency
*f*_0_	free (unloaded) resonance frequency
*F*	contact force
*F_C_*	clamping force
*I*	indentation depth of interest
*I_tot_*	total indentation depth during measurement
n	number of measurements
ν_i_	indentation velocity
|∂F/∂∆f|	stiffness parameter
